# Cyclodextrins for Lithium Batteries Applications

**DOI:** 10.3390/ma16165540

**Published:** 2023-08-09

**Authors:** Mohamed M. H. Desoky, Fabrizio Caldera, Valentina Brunella, Riccardo Ferrero, Gjylije Hoti, Francesco Trotta

**Affiliations:** Department of Chemistry, University of Torino, Via P. Giuria 7, 10125 Torino, Italy; fabrizio.caldera@unito.it (F.C.); valentina.brunella@unito.it (V.B.); riccardo.ferrero@unito.it (R.F.); gjylije.hoti@unito.it (G.H.)

**Keywords:** cyclodextrins, lithium batteries, binders, electrolytes and separators

## Abstract

Due to their high energy and power density, lithium-ion batteries (LIBs) have gained popularity in response to the demand for effective energy storage solutions. The importance of the electrode architecture in determining battery performance highlights the demand for optimization. By developing useful organic polymers, cyclodextrin architectures have been investigated to improve the performance of Li-based batteries. The macrocyclic oligosaccharides known as cyclodextrins (CDs) have relatively hydrophobic cavities that can enclose other molecules. There are many industries where this “host–guest” relationship has been found useful. The hydrogen bonding and suitable inner cavity diameter of CD have led to its selection as a lithium-ion diffusion channel. CDs have also been used as solid electrolytes for solid-state batteries and as separators and binders to ensure adhesion between electrode components. This review gives a general overview of CD-based materials and how they are used in battery components, highlighting their advantages.

## 1. Introduction

The demand for efficient energy-storage solutions has led to an increased interest in lithium-ion batteries (LIBs) as a promising option [1,2]. This is due to their ability to offer high energy and power density, making them ideal for use in a variety of applications, including electric vehicles and hybrid electronic devices [1,2,3,4,5,6,7,8,9,10,11,12,13,14]. After the initial phase of fundamental scientific progress, the late 1960s or early 1970s marked the emergence of primary lithium-ion batteries (LIBs), which eventually paved the way for the first generation of secondary LIBs. In 1972, Matsushita showcased a primary LIB using Li//organic electrolyte//CFx. Around the same time, another researcher named Moser presented a primary solid lithium iodine battery. Following this, a group of researchers including Whittingham, Armand, Goodenough, and Scrosati spearheaded the development of the first rechargeable secondary LIBs [15]. A significant breakthrough occurred in 1991 when Sony Corporation introduced rechargeable LIBs with a LiCoO_2_ cathode and graphite anode to the commercial market. Subsequent improvements in energy density brought the capacity to approximately 155 Whkg^−1^ (400 WhL^−1^), propelling the technology into the second and third generations of rechargeable LIBs. This achievement prompted numerous companies to enter the production arena and support further research for the advancement of secondary LIBs [16]. In addition to these milestones, a noteworthy accomplishment in 1997 was the discovery of the affordable, non-toxic, and environmentally-friendly olivine LiFePO_4_ cathode [17]. These significant advancements ushered in a new era, presenting challenges for LIBs to be incorporated into emerging consumer electronics and potentially other areas as promising environmentally friendly energy storage alternatives to perishable and environmentally harmful fossil fuels [15].

**Figure 1:**
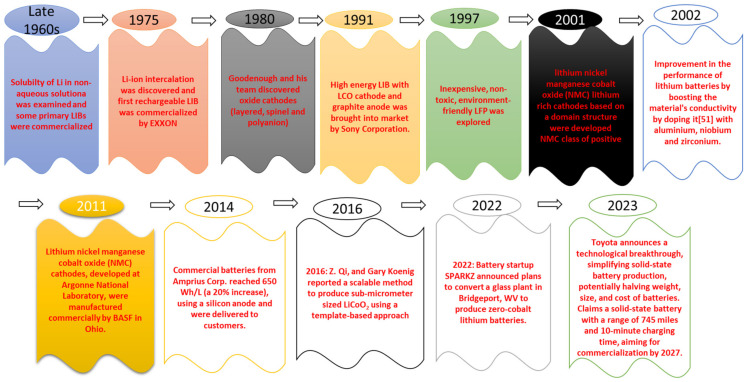
Overtime progress in LIBs.

Figure 1 below provides a timeline-based development chart illustrating the evaluation of early LIBs [6,12,18,19,20,21,22,23,24,25,26,27].

**Figure 2:**
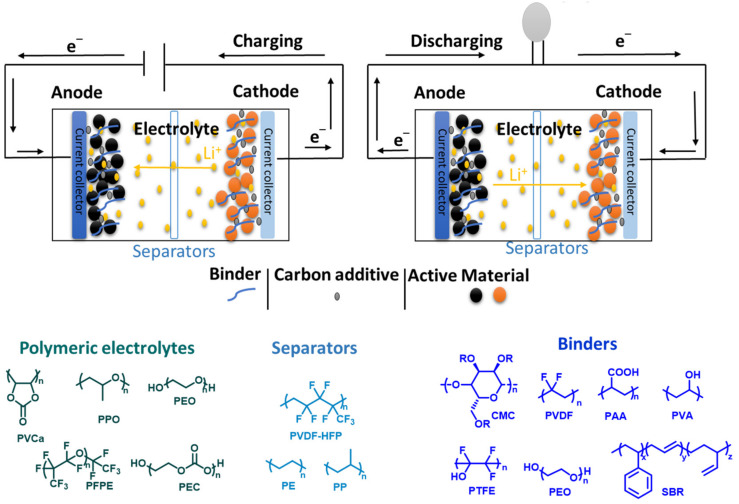
Schematic presentation of a LIB while charging and discharging and summary of polymers used in LIB [3,32].

The fundamental principles of all types of lithium-based batteries are identical, regardless of the electrode material used. During the charging cycle, the Li ions in the cathode material move from the electrode and travel through the separator and electrolyte to arrive at the anode, where they are stored in the structure of the anode material in various ways. When the battery is discharged, the lithium ions leave the anode and travel through the electrolyte to the cathode, where they release energy that powers the battery once more. Electrons are created and compelled to move through the external circuit in the opposite direction of the Li ions during both the charging and discharging cycles. The electrolyte must prevent electrons from passing through, making sure that they instead flow through the external circuit to power an electronic device [3,4,7,8]. Both the flow of electrons through the external circuit and the flow of ions through the electrolyte are interdependent processes, so if one stops, the other also stops. For example, if the Li ions stop circulating in the electrolyte because of a fully discharged battery, the flow of electrons through the external circuit will also stop, causing the battery to stop supplying power to the device. Similarly, the flow of electrons will stop if the battery-powered device is turned off, which will stop the flow of Li ions [3,4,7,8]. The electrode in a lithium-ion battery plays a crucial part in determining its performance [28,29,30]. Thus, optimizing its architecture is a key factor in achieving optimal performance from the battery [31]. In response to the rising demand for batteries with better capabilities, cyclodextrin architectures have been used to improve the performance of Li-based batteries. The goal has been to create new functional organic polymers that can enhance the functionality of already existing materials and facilitate the development of next-generation batteries. The majority of polymers lack electrochemical activity, but whether in liquid or solid form, they are essential to Li-based batteries. These polymers frequently act as solid electrolytes, binders, and separators, giving battery devices mechanical stability, adhesion, and structural support [32,33,34,35,36,37,38,39,40]. Within this context, CD supramolecular chemistry emerges as an ideal engineering framework that enables the incorporation of cutting-edge functionalities into polymeric materials [41,42,43,44,45,46]. Figure 2 depicts an LIB system and shows the most-used polymers for its components. Despite the differences in electrode materials, the basic components and principles of all lithium-ion batteries remain the same.

**Figure 3:**
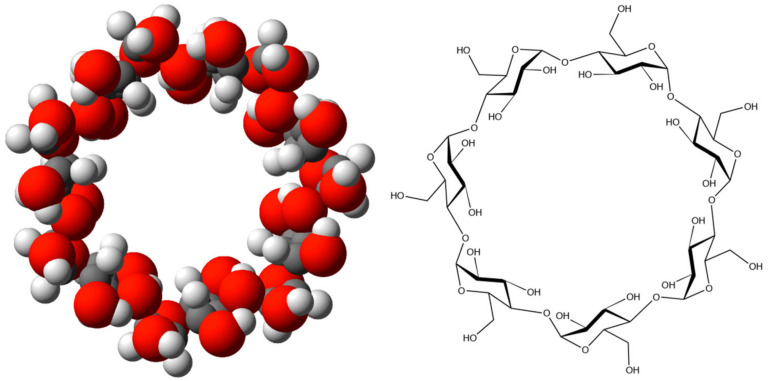
β-Cyclodextrin: Top view of the Stuart molecular model (**left**) and structure (**right**). Reproduced by permission [58].

CDs, or cyclodextrins, emerge as a product when enzymes break down starch, an essential polysaccharide [47,48,49]. These environmentally derived substances belong to the category of cage molecules due to their structurally stable hydrophobic cavity at the center, enabling them to adeptly capture and encase other molecules [50,51]. This exceptional encapsulation property creates a “host–guest” relationship that can change and improve the encapsulated molecule’s physical, chemical, and biological properties. Pharmaceuticals, food, chemistry, chromatography, catalysis, biotechnology, agriculture, cosmetics, hygiene, medicine, textiles, and the environment are just a few of the industries where CDs are widely used [52,53,54,55,56,57]. Despite being discovered 129 years ago, compact discs did not really take off until the 1980s, especially in the food and pharmaceutical industries. This growth was made possible by the large-scale production of three native CDs, namely α-, β-, and γ-CDs, in a highly pure form since 1984. The improved accessibility and affordability, especially of β-CD, played a crucial role in their extensive development (Figure 3) [58].

Cyclodextrins (CDs) are depicted as macrocyclic oligosaccharides consisting of six to eight (1,4)-α-D-glucopyranose units [59]. These amphiphilic macromolecules have relatively hydrophobic cavities and a hydrophilic surface [60]. The unique macrocyclic structure of CDs enables CD-based materials to function as desired carriers or channels at the molecular scale [44]. Solvated lithium ions in the electrolyte are typically considered to have a space size of approximately 0.98 nm [61]. β-CD as the lithium-ion was chosen as a diffusion channel because its inner cavity diameter is approximately 0.88 nm, similar to the size of solvated lithium ions [62]. Additionally, β-CD molecules possess 21 hydroxyl functional groups [58], and the surface of silicon nanoparticles is rich in Si-OH functional groups, enabling strong hydrogen-bonding interactions. Compared to intermolecular interactions, hydrogen bonding is much stronger, allowing β-CD molecules to attach to the silicon surface firmly. Consequently, during the electrode-preparation process, strong supramolecular interactions occur between β-CD and the surface of Si nanoparticles, ensuring the firm adhesion of β-CD to the rough silicon surface. Throughout the lithiation and delithiation process, β-CD molecules contribute to the formation of the SEI layer, providing effective channels for lithium-ion transport. In the development of battery technology, the roles of binders, electrolytes, and separators are crucial for ensuring efficient and safe operation [63]. Binders play a vital role in maintaining strong adhesion to the current collector and cohesion between various electrode components [64,65,66,67]. Traditional binders, such as sodium carboxymethyl cellulose (CMC), poly(acrylic acid) (PAA), poly(vinyl alcohol) (PVA), poly(ethylene oxide) (PEO), poly(tetrafluoro ethylene) (PTFE), poly(vinylidene fluoride) (PVDF), styrene butadiene rubber (SBR), and poly(vinylidene fluoride) have been used to accommodate the unique properties of the active material [68,69,70]. The electrochemical reactions that occur in battery electrodes result in the flow of ions through the electrolyte and electrons through the external circuit. In liquid-based batteries, separators play a critical role in electrically isolating the two electrodes while allowing ion conduction through their pores. These pores typically range in size from 30 to 100 nm. Common materials used in separator construction include poly(vinylidene fluoride-co-hexafluoropropylene) (PVDF-HFP) and polyolefins like poly(ethylene) and poly(propylene) (PE) [71]. To enhance battery safety by reducing the risk of fire caused by liquid electrolytes, liquid Li-ion batteries are being transformed into solid-state devices [72,73]. This transition necessitates the development of solid electrolytes. Solid polymer electrolytes (SPE), which involve dissolved Li-salts in a polymer matrix, have gained significant attention in research. While poly(ethylene oxide) (PEO) remains the most widely used polymer for this purpose, other polymers, including poly(propylene oxide), poly(tetrahydrofuran), poly(ethylene carbonate), poly(vinylene carbonate), and perfluoropolyether (PFPE) have been explored [74]. Recently, the intriguing CD host–guest interaction has been used to create cutting-edge polymer-based battery components, including binders, separators and specialized electrolytes. This strategy has enabled the achievement of several desirable properties, including (i) the customizing of electrolytes, whether in liquid form or as solid polymer electrolytes (SPE), to improve the performance of advanced Li-ion batteries; (ii) the development of efficient binders for silicon (Si) anodes, etc.; (iii) the development of separators. The benefits of CD host–guest chemistry in the LBs are covered in detail in the following sections.

## 2. Cyclodextrin for Lithium-Battery Improvements

Cyclodextrin structures have emerged as a promising solution to enhance the performance of Li-based batteries. The increasing demand for more efficient batteries has driven the creation of novel organic polymers, aiming to optimize existing materials and facilitate the development of next-generation battery technologies. While many polymers lack electrochemical activity, they are widely used in Li-based batteries, whether in liquid or solid form, serving as binders, separators, and solid electrolytes. These polymers play crucial roles in providing mechanical stability, adhesion, and structural support to the battery devices. In this context, the supramolecular chemistry of cyclodextrins offers an excellent platform for engineering advanced functionalities into polymeric materials, presenting new opportunities for battery technology improvement.

### 2.1. Cyclodextrin-Based Binders

#### 2.1.1. Anode Binders

**Figure 4:**
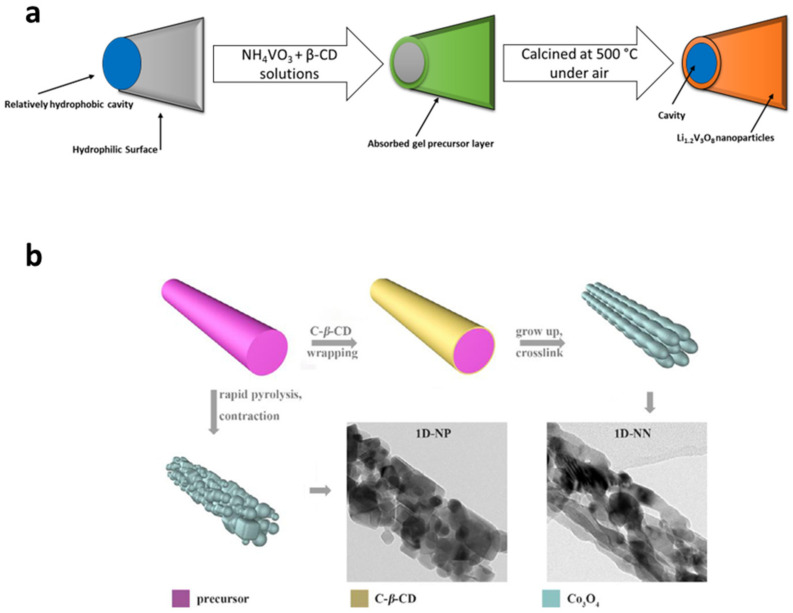
(**a**) Formation mechanism of a single macaroni-like Li_1.2_V_3_O_8_ [87] and (**b**) 1D-NN and 1D-NP, reproduced by permission [88].

Silicon (Si) has emerged as a highly promising candidate for advanced Li-ion battery-anode materials due to its impressive theoretical capacity of 3600 mAh·g^−1^, nearly ten times higher than commercialized graphite anodes (372 mAh·g^−1^). Additionally, Si is abundant in nature, cost-effective, and exhibits a low discharge voltage (<0.5 V vs. Li^+^/Li). However, Si faces significant challenges during the electrochemical alloying processes of lithiation (charge) and delithiation (discharge). These processes lead to considerable volume fluctuations (~300%), causing the structural collapse of the electrode. As a result, the Si electrode experiences cracking, pulverization, and delamination, leading to the electrical isolation of certain Si domains. Another critical concern is the stability of the solid electrolyte interface (SEI), which forms at the electrode–electrolyte interface as a layer composed of electrolyte-reduction products. This layer continuously grows as new Si surfaces are created. Unfortunately, these degradation mechanisms significantly impact the cycling performance of Si anodes, resulting in poor cycling capabilities. As a result, a substantial amount of research is dedicated to enhancing the cyclability of Si electrodes. Much work has been done on the design of Si anode binders that can preserve the electrode structure and accommodate mechanical stress during cycling [75,76,77]. Among the Si anode binders commonly used are synthetic and bio-derived polymers containing carboxyl and hydroxyl groups, such as poly(acrylic acid) (PAA), ref. [78] sodium alginate, ref. [79] and sodium carboxymethyl cellulose (CMC) [80]. These polymers exhibit spontaneous interactions with the Si surface (Si-OH) through hydrogen bonding, allowing for reformation if locally broken under stress, resulting in a self-healing effect. Considering the chemical similarities between CD, PAA, CMC, and alginate, the use of CD-based binders is a plausible approach. The self-healing and mechanical properties derived from the CD host–guest interactions have inspired the engineering of new Si anode binders that can mimic the “breathing” behavior of Si electrodes during cycling, involving expansions and contractions. A research group has proposed two main designs for such binders [81,82]. The first design, known as the side-chain-complex approach, involves host–guest cross-linking between a hyperbranched polymer prepared from β-CDs (host-polymer) and a dendritic modified gallic acid containing six adamantane moieties (guest-crosslinker) [81]. The 3D structure of the β-CDs hyperbranched polymer has been shown to reinforce mechanical properties and facilitate the recovery of broken linkages (Si-binder and binder-binder interaction) [83]. Furthermore, the addition of dynamic host–guest cross-linking promotes the self-healing ability. Importantly, the stronger binding of β-CD to adamantane at the molecular level has a direct positive impact on cycling performance, as evidenced by poorer results obtained with analogous α- and γ-CDs hyperbranched polymers. Overall, this binder design has proven effective in improving the cyclability of Si electrodes compared to PAA, CMC, or alginate binders. The second example pertains to the main-chain-complex approach, offering a novel perspective on the CD host–guest interaction. Drawing inspiration from the principle of moving pulleys, Choi et al. [82]. introduced a groundbreaking topological cross-linking concept. In this approach, poly(acrylic acid) (PAA) is cross-linked to α-CDs-based pseudo-rotaxanes (PRs), resulting in a mechanically interlocked network known as PR-PAA. This network features movable cross-links, enabling PR-PAA networks to withstand significant strains (up to 390%) and exhibit superior stress–strain recovery properties compared to PAA. The enhanced mechanical strength of PR-PAA is attributed to the sliding motion of CDs along the threading polymer. Moreover, as a binder, PR-PAA outperformed PAA in terms of capacity retention, and microscopic analyses revealed reduced cracking in the Si particles. These impressive capacity retentions have spurred further research, and the topological cross-linking concept was combined with boronic esters (reversible covalent bond) to confer high elasticity and self-healing properties to the Si anode binder [84]. Recent extensions of the PR-PAA approach include its application to silicon monoxide (SiO) electrodes [85] and carbon nanotubes, both of which are considered promising mechanically stable lithium hosts for Li metal anode development [86]. In 2010, C. Cheng et al. prepared novel macaroni-like nanoparticles based on Li_2_V_3_O_8_ through complexation with β-cyclodextrin (β-CD) to be utilized as an anode for aqueous rechargeable lithium-ion batteries (ARLB), as shown in Figure 4 [87]. The ARLB was built using LiMn_2_O_4_ cathode, 1.0M Li_2_SO_4_ electrolyte, and the absorbing complex with β-CD as the anode. The initial capacities of the anode material gave 189, 140, and 101 mAhg^−1^ at 0.1, 0.5, and 1.0 C, respectively. By combining the solution of the NH_4_VO_3_ and the Li_2_CO_3_ with β-CD solution leading to absorption of NH_4_VO_3_ and Li_2_CO_3_, a nanoparticle resembling macaroni is created. The majority of β-CDs form micelles that resemble gel precursors in the mixed solution as the water is being evaporated. Then, the micelles gather and absorb water, forming the precursor of Li_1.2_V_3_O_8_. To form the macaroni-like nanoparticle, the Li_1.2_V_3_O_8_ precursor is calcined in an atmosphere of 500 °C, leaving a cavity. In 2007, Yongyuan Hu et al. reported a one-dimensional Co_3_O_4_ nanonet for lithium-ion batteries with an improved rate of performance by studying the effect and kinetics of introducing C- β-CD as a cross-linking agent [88]. C- β-CD was used as a crosslinking agent (Figure 4), resulting in the interconnection of the independent nanoparticles. This new cross-linking material based on C- β-CD was utilized as the anode in a lithium-based battery displaying a superior rate capacity of 982 mAh/g at a discharging current density of 2000 mA/g. They used GiTT, EIS, and CV to study the kinetic properties, and these electrochemical techniques proved that the C- β-CD-based anode is conductive to Li^+^/electron transfer, which is a result of the more interior transportation routes. As a result, Hu et al. show that C- β-CD 1 D nanonet could be used as an anode layer for high-performance lithium-based batteries.

**Figure 5:**
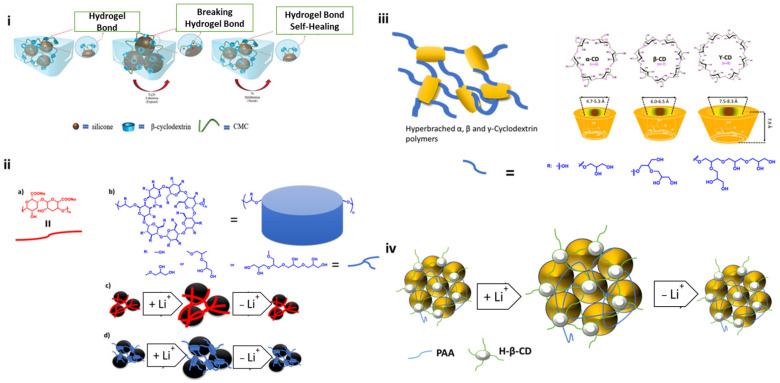
(**i**) Interaction mechanism between the Si particles and the binder. Reproduced by permission [89]; (**ii**) (**a**,**b**) graphical representation and chemical structure of (**a**) the alginate(Alg) and (**b**) β-CDp binders; (**c**,**d**) representation of Si-binder structure for (**c**) Sialg and (d) Siβ-CDp through lithiation/dilithiation of Si [83]; (**iii**) graphical representation of the CDs polymers and chemical structures of cyclodextrins and linkers [81]; (**iv**) the interaction among PAA-β-CDp crosslinking binder and Si atoms [90].

In 2021, Hao-wen Jiang reported that the advantages of the 3D network structure created by the cross-linked binder’s two distinct components prove to be highly favorable for Si negative electrode materials (Figure 5) [89]. This intricate three-dimensional framework successfully withstands the considerable volume fluctuations experienced by Si during the charging and discharging processes. The binder’s reactive groups (hydroxyl, carboxyl) establish stable chemical bonds with Si, ensuring the electrode’s structural stability over multiple cycles, ultimately leading to an impressive reversible capacity. There are several key reasons why the three-dimensional structure of β-CD-CMC is of utmost importance in optimizing the performance of silicon-based electrodes during cycling. Firstly, β-CD-CMC inherits CMC’s adhesion capability while possessing its unique three-dimensional structure, thereby enabling superior long-term cycling performance. Secondly, the incorporation of β-CD in β-CD-CMC contributes to the partial stabilization of the SEI layer formed by silicon particles during charge and discharge. Lastly, the presence of hydrogen bonding within β-CD-CMC facilitates a certain degree of self-repair for the cracks that emerge in the electrode during cycling. The Si/β-CD-CMC electrode exhibits a remarkable reversible capacity of 1702 mA h g^−1^ even after 200 cycles under high current conditions (0.5 C), surpassing the performance of both Si/β-CD and Si/CMC electrodes. Consequently, β-CD-CMC stands out as an effective and environmentally friendly binder for Si-based electrodes, providing a promising avenue for future industrial advancements. In 2014, a hyperbranched β-cyclodextrin polymer (β-CDp) was reported by You Kyeong Jeong et al. as an efficient multidimensional binder for silicon anodes in lithium rechargeable batteries (Figure 5) [83]. As a binder for Si-based anodes in lithium-ion batteries, they introduced a multidimensional hyperbranched β-CD polymer. The distinctive β-CD macrocycle structure and its three-dimensional polymerization led to a hyperbranched polymeric network which enhances the interactions of Si-binders and the self-healing properties, improving the mechanical stability of the electrode layer and leading to overcoming the chronically inadequate cycle lives of the Si-based anode. After 200 cycles, the specific capacity of Siβ-CDp was 1471 mA g^−1^, retaining up to 68.7% of its initial value of 2142 mA g^−1^. Siβ-CD, in contrast, had a dramatic decrease in its specific capacity to 460 mA g^−1^ after 200 cycles, starting at a lower capacity of 1810 mA g^−1^, retaining up to 25.4% of its capacity.

In 2015, Tae-woo Kwon et al. reported supramolecular cross-linking polymers based on CDs (Figure 5) [81]. According to their control inclusion experiment using an adamantane unit and α, β, and γ CDs, it was proven that βCD-based polymer showed the best life cycle because of the inclusion interaction between adamantane and β-CD, the host. At the same level of active material loading, this dynamic cross-linking results in superior cycling performance compared to that of host or guest alone as well as most other traditional binders due to excellent Si-binder interactions, the good structural stability of electrode film, and controlled SEI formation. Without a dynamic cross-linker, all three types of CD polymers based on α, β, and γ showed comparable capacity retentions after 150 cycles of 64%, 69%, and 53%, respectively. The dynamic cross-linking enables close interactions between the silicon binder, ensuring structural stability of the electrode film and controlled SEI (Solid Electrolyte Interphase) formation. As a result, this leads to significantly improved cycling performance compared to using either the host or guest alone, as well as surpassing the performance of most conventional binders when the same level of active material loading is employed (Table 1). In 2022, Song Lin et al. displayed new cross-linking binders (hyperbranched polymers) based on polymerized β-Cyclodextrin (β -CDp) and polyacrylic acid (PAA) that can improve the capacity performance of Si/C composite in lithium ion batteries (Figure 5) [90]. The PAA-β-CDp showed a 3D network structure, which provides a strong adhesion between the current collector and the active material, as well as mechanical stability of the electrode through self-healing properties due to the hydrogen bonding. The cross-linked polymer β-CDp was synthesized using the cross-linking agent epichlorohydrin (ECH) to β-CD. Moreover, the addition of the ECH cross-linking unit influenced the β-CDp polymer’s ability to dissolve in water. β-CD:ECH = 1:14, the maximum cross-linking degree with the best water solubility, is considered the best cross-linking ratio for the optimal cycling stability. The linear PAA polymer’s mechanical strength is increased with the addition of the cyclic 3D -CDp, making the resulting electrode more durable and cycling-efficient. The highest cycling performance of the electrode is presented by a ratio of 8:2 of PAA: β-CDp. This device with the Si-PAA(8)-CDp(2) electrode displayed a stable capacity of 2326.4 mAhg^−1^ at 0.2 Ag^−1^, after 100 cycles, with a capacity retention of 64.6% and good stability. The simplicity of the preparation of the Si-PAA(8)-CDp(2) and the low cost of the raw materials lead to the development of silicon-based anodes for lithium ion-based batteries.

Although these approaches seem elegant and promising, there is still a lack of thorough scientific investigation. While some studies have investigated the performance of host–guest cross-linking binders based on factors like CD type, binder loading, and guest cross-linker nature, we still have no clear understanding of how the structural parameters affect the topological cross-linker.

#### 2.1.2. Cathode Binders

**Figure 6:**
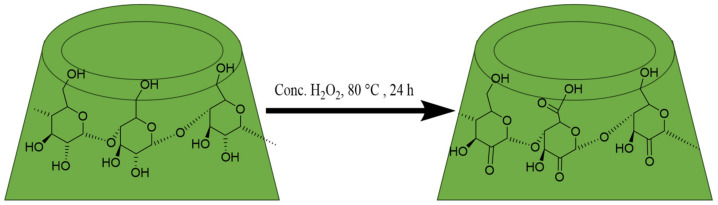
Reaction of β-CD with H_2_O_2_.

**Figure 7:**
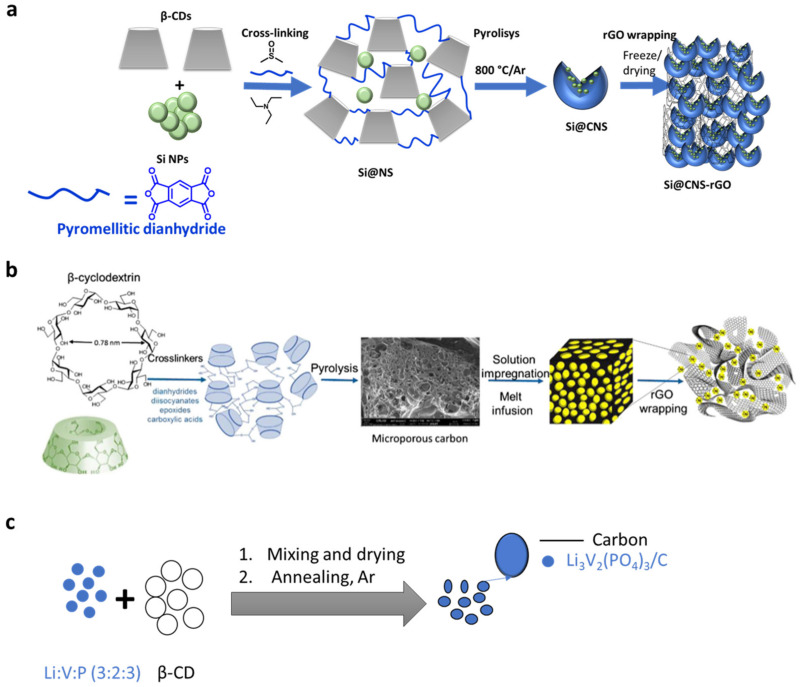
*(***a**) Synthesis steps of Si@CNs-rGO [101] and (**b**) the method for synthesizing functional microporous C/S wrapped with reduced graphene oxide (rGO), reproduced by permission [100] and (**c**) synthesis process of Li3V2(PO4)3/C [102].

Preventing the solubilization of redox active species is crucial for the successful implementation of Li-S batteries [91]. These batteries, comprising a Li metal anode and a sulfur (S) cathode, offer high theoretical specific energy (2600 Wh·kg^−1^), far exceeding conventional Li-ion batteries with transitional metal oxide cathodes and carbon-based anodes (~300 Wh·kg^−1^). Despite the promising features of S, including its abundance, low cost, and eco-friendliness, certain technical challenges have hindered practical applications of Li-S batteries. The main obstacle arises during discharge, where S is reversibly converted into Li_2_S through a multistep redox process, generating soluble lithium polysulfide intermediates with various chain lengths (Li_2_S_x_ with x = 2–8) [92]. These polysulfides tend to migrate back and forth between the electrodes, giving rise to the “polysulfide redox shuttle” [93]. The overall reduction at the anode results in a poorly controlled Li/electrolyte interface due to the deposition of insoluble Li_2_S. This phenomenon leads to low utilization of the active material and consequently poor electrochemical performance of Li-S batteries [94]. Considerable progress has been made to address the issue of persistent polysulfide migration. This includes designing entangled separators with porosity, trapping, and repulsive interactions, developing novel materials such as electrolytes, and exploring new cell configurations. Special attention has been given to sulfur encapsulation techniques at the positive electrode to limit polysulfide dissolution through surface adsorption on oxides, mesoporous carbon confinement, and the use of “wrapping” binders. Efficient cyclodextrins (CDs)-based binders have been reported in this context [95,96,97]. The following section aims to assess the impact of CD molecular recognition properties towards polysulfides on the efficiency of these reported CDs-based binders. Firstly, an oxidized β-CD derivative was found to be a viable aqueous binder, outperforming commonly used PVDF and PTFE binders. The superior water solubility of the β-CD derivative contributed to better cycling performance by ensuring good surrounding of the S particles during the slurry process. Subsequently, incorporating a β-CD hyperbranched polymer in the S-based cathode further enhanced Li-S battery performance due to the excellent wrapping properties of the 3D polymer architecture. Moreover, a strategy to immobilize polysulfides involved grafting cationic moieties (quaternary ammonium cations) onto the hydroxyl groups of β-CDs in the hyperbranched polymer. The attractive electrostatic interactions between the negatively charged polysulfides and the cationic CDs-based binder led to improved cycling performance. However, the use of CDs in these applications has remained somewhat empirical, lacking clear rationalization of the role played by CD cavity dimensions concerning the size of the polysulfide intermediates. This observation should spark the researchers to explore the potential host–guest interactions of CDs with polysulfides and should open the door for future investigations [98,99]. The use of carbonyl β-CD as a new binder for S-based cathode in rechargeable LIBs was reported by Jiulin Wang et al., 2013 [96]. They obtained carbonyl β-CD derivatives by partial carbonylation of the hydroxyl group with H_2_O_2_ (Figure 6); the obtained C-β-CD showed 100 times higher solubility in water than that of β-CD at ambient temperature. Significantly, C-β-CD demonstrated a strong bonding ability that is not exhibited by β-CD and electrochemical stability between 0 and 5 V, making C-β-CD a suitable match for the binder in battery technology. The electrochemical performance of the sulfur-based composite using C-β-CD as a binder revealed a higher reversible capacity of 694.2 mA h g_(composite)_^−1^ and 1542.7 mA h g_(sulfur)_^−1^, the utilization of sulfur approaching 92%; cycling performance was also superior. When C-β-CD was used as the binder, the sulfur cathode’s discharge capacity was 1456 mA h g_(sulfur)_^−1^ after 50 cycles, which was higher than that of cathodes using β-CD. Due to the strong bonding and high solubility of C-β-CD, after the cathode has been fried, a gel film forms and tightly warps the surface of the sulfur composite, preventing it from aggregating. Excellent cycling performance was noticed due to the stable homogeneous distribution of the cathode structure and the sulfur composite throughout the charge-discharge cycle. In addition, C-β-CD is superior to traditional PVDF and PTFE binders because it has high electrochemical stability, reduced cost, and environmental protection. According to the authors, in addition to the sulfur cathode in a rechargeable lithium battery, C-β-CD based materials are promising binders for other electrode materials used in lithium-ion batteries. In 2017, βCD-based NSs were used by Usman Zubair et al. to warp the dual confinement of sulfur with an rGO cathode layer to use in Lithium/Sulfur based batteries (Figure 7) [100]. First, they crosslinked the β-CD to create microporous carbon spheres with pores ranging from 5 to 11°A. Then, they complexed the carbon with sulfur, using solution impregnation and melt infusion. Finally, they wrapped the C/S complex in reduced graphene oxide (rGO) to create conductive pathways to the sulfur cathode and to safeguard the surface-adhered sulfur. The resulting cathode has a capacity for initial discharge of 1103 mA h g^−1^ at 0.1 C and 626 mA h g^−1^ at 0.2 C. The losing capacity was at a rate of 0.2% per cycle for cycles greater than 100. By the introduction of a commercially available CFP interlayer to the β-CD sulfur-based cathode, the capacity increased to 850 mA h g^−1^ at 0.2 C, with decay less than 0.14% for cycles greater than 100.

**Figure 8:**
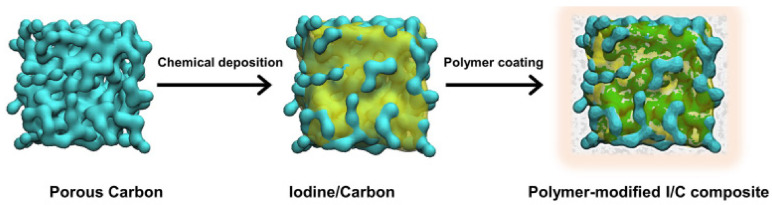
Illustration showing the process of fabricating polymer-modified I_2_/AG composites. Reproduced by permission [105].

In 2016, Chen et al. introduced a novel cathode material for lithium-ion batteries (LIBs) by coating lithium vanadium phosphate with β-cyclodextrin (β-CD) [102]. The Li_3_V_2_(PO)_3_/C cathode layer was prepared using a rheological phase method (Figure 7). X-ray diffraction (XRD) analysis confirmed a pure monoclinic structure with sharp diffraction peaks, indicating the material’s crystallinity. Scanning electron microscope (SEM) and transmission electron microscope (TEM) imaging revealed uniformity and optimal size, which played a crucial role in the electrochemical behavior of LIBs. The initial discharge capacity was 111 mA h g^−1^ and remained at 109.3 mA h g^−1^ after 50 cycles at 0.1 C rate in the voltage range of 3.0–4.3 V. At a higher voltage range of 3.0–4.8 V, the initial discharge capacity was 151.4 mA h g^−1^ after 25 cycles at 0.1 C. This demonstrated the promise of β-CD as a carbon source for LIB materials. In 2018, Liu et al. reported the synthesis of NH_4_V_4_O_10_ nanoflowers using a simple hydrothermal process with β-cyclodextrin as a flexible template [103]. These nanoflowers, composed of 200–300 nm-sized nanoflakes with distinct outlines and uniform sizes, contained mesopores with an average size of 3 nm. This straightforward method could be applied to prepare other three-dimensional ammonium vanadate nanomaterials. The β-CD-based cathode nanoflowers exhibited a superior specific capacity of 242.8 mAh g^−1^ at 200 mA g^−1^ current density in the voltage range of 2.0–4.0 V, demonstrating excellent cycling stability. They retained about 64.9% of the specific capacity (103.5 mAh g^−1^) at 1000 mA g^−1^, making NH_4_V_4_O_10_ β-CD-based nanoflowers promising cathode materials for lithium-ion batteries. In 2018, Cai et al. presented an iodine/β-CD-based cathode for lithium-iodine battery applications [104]. This cathode was synthesized by utilizing iodine and β-CD through a facile saturated water solution procedure. The relatively hydrophobic cavity of β-CD effectively encapsulated the iodine molecules, preventing their dissolution within the electrode. The battery with this iodine/β-CD cathode exhibited an initial specific charge capacity of 175 mAh g^−1^ at 0.1 C current density, retaining around 94% (164 mAh g^−1^) after 300 cycles. These findings demonstrated that β-CD serves as an excellent host for encapsulating iodine molecules, enhancing the stability of the iodine-based cathode, and showcasing the potential for low-cost and facile preparation of cathodes in lithium-iodine batteries. In 2020, Zhang et al. [105] presented a novel approach to enhance the cycling performance of iodine-based cathodes by encapsulating iodine molecules in methyl-beta-cyclodextrin (Mβ-CD). To achieve this, they developed three nonionic water-soluble polymers based on polyvinylpyrrolidone (PVP), amylose corn starch (ACS), and Mβ-CD to coat iodine/carbon with a high concentration of iodine (Figure 8). By using Mβ-CD, PVP, and ACS in the batteries, they observed discharging capacities of 227.7 mAh g^−1^, 222.1 mAh g^−1^, and 224.1 mAh g^−1^ at the second cycle, respectively. The capacity retention after 500 cycles was found to be 87.3%, 90.1%, and 79.7% when using Mβ-CD, PVP, and ACS, respectively. Furthermore, the coulombic efficiencies of the batteries remained above 97%, 98%, and 96% after the initial cycle, respectively, in the presence of Mβ-CD, PVP, and ACS. This study demonstrates a promising technique to prepare high-performance electrodes with highly active materials for lithium-iodine batteries. The use of cyclodextrin, especially Mβ-CD, provides an effective solution to enhance the cycling stability and efficiency of the iodine-based cathodes, contributing to the development of improved lithium-iodine battery technology.

#### 2.1.3. Anode and Cathode Binders

**Figure 9:**
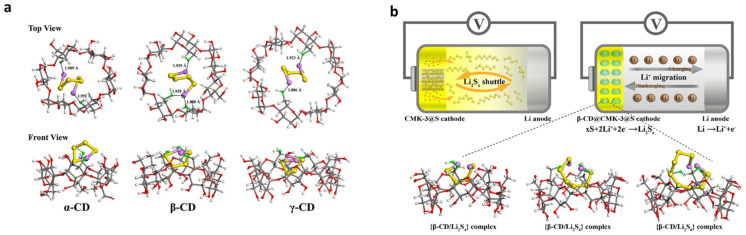
(**a**) Binding connectivity between cyclodextrin (CD) hosts and Li_2_S_4_ guests shown in two views. Colors represent different atoms: C (gray), H (light gray), O (binding oxygen, red/green), Li (purple), and S (yellow); and (**b**) The trapping behaviors of OH-ordered structures. β-CD modification effectively captures dissolved lithium polysulfides within its internal cavities. Colors represent atoms: C (gray), H (light gray), O (binding oxygen, red/green), Li (purple), and S (yellow). Reproduced with permission [106].

In 2020, Ren et al. conducted experimental and calculated investigations using cyclodextrins (CDs) as a model platform to study the trapping mechanism of lithium polysulfides (Li_2_S_x_) (Figure 9) [106]. They discovered that different CDs exhibit varying trapping performances with Li_2_S_x_ species, with the trapping efficacy following the order α-CD < γ-CD < β-CD. The density functional theory (DFT) studies revealed the significant role of the asymmetrical arrangement of primary hydroxyl groups in CDs, which proved to be a crucial factor in determining the trapping density. Electrodes incorporating β-CD demonstrated improved electrochemical performance by effectively restricting the diffusion of high-order polysulfides into the electrolyte. Moreover, β-CD enhanced the movement of lithium ions within the sulfur cathode, leading to a twofold increase in available capacity. The use of cyclodextrin, particularly β-CD, as a trapping agent offers significant benefits, including enhanced trapping efficiency for Li_2_S_x_ species and improved optical performance in the lithium-sulfur battery. By addressing the trapping issues associated with polysulfides, β-CD helps to optimize the battery’s performance and increase its available capacity. This research provides valuable insights for the development of more efficient and high-performing lithium-sulfur batteries in the future. In 2022 Mojtaba et al. prepared micro-mesoporous carbons-based βCD NSs with the improved capacity of Si anodes and S cathodes for LIB based on Si and S [101]. New carbons are made from NSs that are biobased to host S and Si nanoparticles to obtain high-capacity electrodes that can be utilized in lithiated Si-S devices, which are synthesized by taking advantage of having the same carbon matrix for both (Figure 7). In this investigation, for the first time, an effective template method for creating mesopores in the nanosponge, leading to the absorption of sulfur within the carbon, was demonstrated. This spongy structure enhances the process. The preparation of the carbon nanosponges (CNS) is facile to scale up and discover new methods for mesoporous carbon synthesis. The initial cell capacity is 927 mAhg^−1^ at 0.2 C and retains 85% over 100 cycles, proving improved cycling stability.

### 2.2. Cyclodextrin-Based Electrolytes

**Figure 10:**
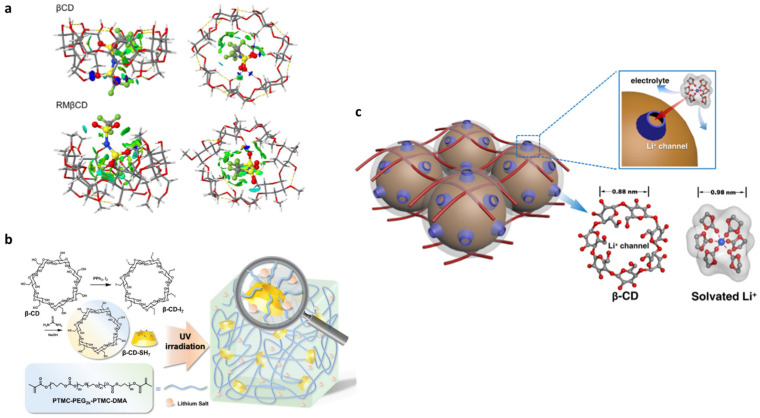
(**a**) Structures of TFSI inclusion in β-CD and RM β-CD are displayed, showcasing intramolecular (yellow) and intermolecular (blue) hydrogen bonds. NCI surface representation highlights strong attractive interactions (blue) and van der Waals interactions (green); (**b**) cyclodextrin-based triblock polymer electrolytes; (**c**) representations of β-CD as the lithium-ion diffusion channel, and the size ofβ-CD and solvated lithium ion. All reproduced by permission [62,111,114].

**Figure 11:**
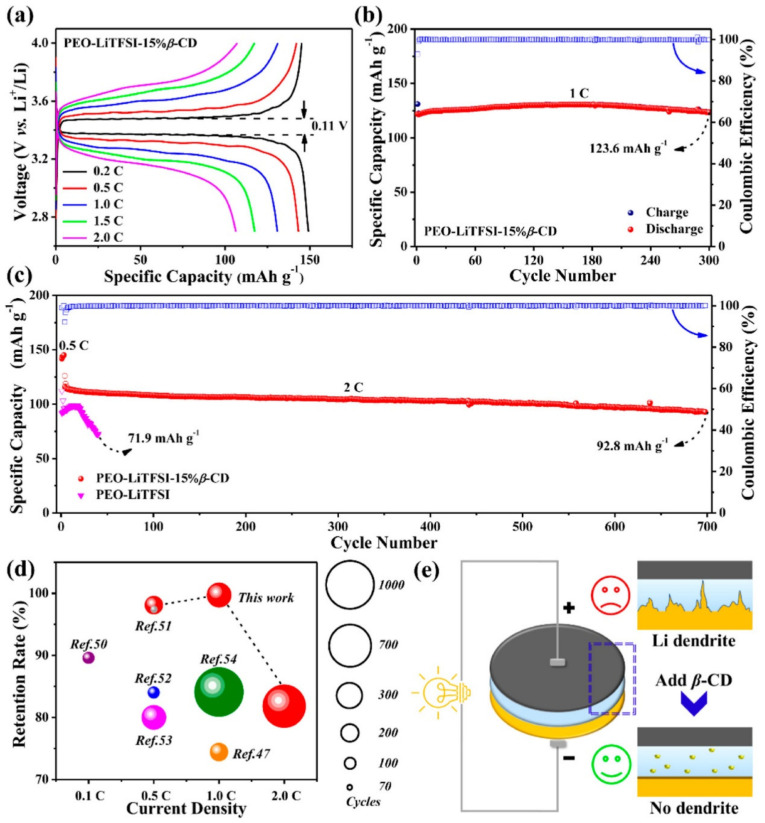
Electrochemical performance of LiFePO_4_/PEO–LiTFSI-15% β-CD/Li cells at 60 °C. GCD curves: (**a**) cycling performance at 1 C rate; (**b**) long-term performances with PEO–LiTFSI-15% β-CD CSE and PEO–LiTFSI SSE at 2.0 C; (**c**) comparison of electrochemical performances; (**d**) schematic illustrating inhibition of lithium dendrites with β-CD in PEO-based SSE (**e**). Reproduced by permission [114].

The structure of Li-based electrolytes [74] has remained largely unchanged since the introduction of the first Li-ion cell to the market by Sony in 1991. These liquid electrolytes typically consist of a lithium (Li) salt dissolved in a mixture of organic solvents. The key property of these electrolytes is their ionic conductivity (*σ*), which determines the mobility of Li ions participating in the electrochemical reactions at the electrodes. Achieving stable electrolytes with a wide potential range (0–4 V) and good interfacial stability with both the anode and cathode is essential [107,108]. In the case of liquid electrolytes, various factors influence ionic conductivity, including the solvent’s dielectric permittivity εR (which affects salt dissolution), viscosity η, glass transition, melting, and boiling temperatures (which facilitate ion transport), as well as interactions between anions, solvents, and Li^+^ ions. To model ion transport in solid polymer electrolytes (SPE), researchers often use the Vogel-Tammann-Fulcher (VTF) model, which relies on the correlation between ionic conductivity and polymer chain motion [108,109]. Two recent strategies have been developed to enhance Li transport properties in liquid SPE electrolytes, both based on cyclodextrin (CD) host-guest interactions. The first approach involves immobilizing the TFSI anion using randomly methylated β-CD (RMβ-CD) to increase Li^+^ contribution to the ionic conductivity. However, dynamic guest-exchange in RMβ-CD/TFSI complexation limited the anticipated increase in TFSI ion mobility, requiring more efficient trapping methods. The authors propose chemical modifications and grafting of the host molecule on immobilized battery components like the separator as potential solutions [110]. The second strategy addresses the challenge of balancing mechanical strength and ionic conductivity in solid-state polymer batteries. Winter’s group introduced a new class of SPEs prepared from CDs-based polypropylene (PPR) or polyrotaxanes (PR) mixed with LiTFSI. The architecture of these electrolytes can be tailored by varying the CD cavity dimensions (α-, β- to γ-CD) or the polymer backbone (nature, molecular weight) and functionalizing the threaded CDs. The conductivity of these electrolytes is influenced by the ring size of CDs, with larger CDs showing higher conductivity. Modifications, such as methylation of PPRs or grafting of poly(ε-caprolactone) flexible side chains on γ-CDs, have shown promising results with enhanced ionic conductivity and improved cycling performance at higher temperatures compared to traditional PEO-based electrolytes [111,112]. Both approaches offer innovative concepts for customizing Li-based battery polymer or liquid electrolytes using CD-based supramolecular chemistry. However, their real performance benefits need further validation. The first approach’s dynamic nature of CD host–guest interaction equilibrium complicates efficient trapping strategies in bulk solutions. The second approach relies on tailored hyperbranched architectures achieved by modifying threaded CDs using flexible scaffold macromolecules, but the influence of CD sliding motion on electrolyte properties requires more investigation. Previous studies have suggested that tubular structures may provide directional pathways for fast Li^+^ transport while restricting anion access through size exclusion, but molecular dynamics simulations have shown that Li^+^ ions are mainly distributed outside the channels [111,112]. In the first strategy, the researchers aimed to address the challenge of capturing the bis(trifluoromethylsulfonyl)imide (TFSI) anion in a conventional salt-in-solvent Li-S battery or lithium-ion battery (LIB) electrolyte [110]. They found that RMβCD (randomly methylated β-cyclodextrin) could serve as a suitable host for encapsulating the TFSI anion, exhibiting similar interaction energy to its non-methylated β-CD counterpart, as revealed by semi-empirical quantum-mechanical (SQM) computational methods which are shown in Figure 10. One of the benefits of using RMβCD is its solubility in the organic solvents PC (propylene carbonate) and DME (dimethoxyethane) commonly used in batteries. The conductor-like screening model for the real solvent (COSMO-RS) method confirmed this solubility. 1D and 2D 1H NMR measurements showed that RMβCD can enclose PC within its hydrophilic environment. However, the researchers observed that the RMβCD-TFSI complex was not formed, likely due to the lack of an entropy-driven process during the ligand-exchange reaction, where the solvent inside RMβCD’s cavity is replaced by TFSI. Nevertheless, in DME and MeOH (methanol), the RMβCD-TFSI complex was successfully formed, as evidenced by ^19^F NMR and FTIR spectroscopy. The Gibbs free energy of formation of this complex was found to be consistent with values observed for other complexes based on cyclodextrins (CDs). Importantly, the study demonstrated that the RMCD-TFSI complex remains mobile within the electrolytes, and the guest exchange dynamic is maintained. This indicates that the presence of the complex does not negatively impact the mobility of TFSI ions, a crucial aspect for effective battery performance. In 2021, Zuo et al. developed new electrolytes consisting of an elastic cyclodextrin-based triblock polymer to be utilized in solid-state lithium-metal batteries to overcome the use of liquid electrolytes, which often encounter issues such as lithium dendrite growth, as well as safety concerns such as leakage and volatilization [113]. The triblock polymer based on cyclodextrin has been synthesized using ring-opening polymerization and initiator-free thiolene reaction, as seen in Figure 11, for application in all-solid-state lithium-ion batteries. This material exhibits high elasticity and stability when used with a metallic lithium anode. The elasticity of the polymer electrolyte is induced by the PTMC unit. It has been established that the incorporation of CDs led to a more uniform deposition of lithium in electrochemical processes. The lithium batteries have demonstrated stable cyclic performance and reversible specific discharge capacity. This work provided a new method for making stable lithium metal batteries. In 2021, Hyanhuan Duan and colleagues introduced an innovative approach for developing highly stable and high-rate all-solid-state lithium-metal batteries by integrating cyclodextrin into poly(ethylene oxide) (PEO)-based electrolytes [114]. They demonstrated that incorporating β-cyclodextrin (β-CD) as a filler in the PEO-based solid-state electrolytes (SSE) significantly enhances both mechanical and electrochemical performances. The use of β-CD as a filler brings multiple advantages. It effectively improves the transfer of Li^+^ ions and enhances the conductivity of the PEO-based SSE. Moreover, hydrogen bonds are formed between the hydroxyl groups of β-CD and the poly(ethylene oxide) (PEO) units, further boosting the mechanical strength of the electrolyte and inhibiting the growth of dendrites on the lithium anode. The all-solid-state lithium batteries (ASSLBs) constructed with the PEO-LiTFSI-15% β-CD composite solid electrolyte (CSE) displayed outstanding electrochemical performance. They achieved an improved specific capacity of 123.6 mAhg^−1^, with 100% capacity retention at 1 C rate, and showed stable cycling over 700 cycles at 2 C rate, as shown in Figure 10. These results highlight the potential of cyclodextrin-integrated PEO-based electrolytes as a promising solution for ultra-stable and high-rate all-solid-state lithium-metal batteries.

In 2021, P. Li et al. [62] addressed the challenges associated with using silicon (Si) as an anode material in lithium-ion batteries. While Si has an incredibly high theoretical capacity, its commercialization is hindered by significant volume changes and the simultaneous formation of a solid electrolyte interphase (SEI) during charging and discharging processes. To overcome these limitations, the researchers introduced a solution using β-cyclodextrin (β-CD) as a lithium-ion diffusion channel at the molecular level (Figure 11). The design of the molecular channel in β-CD matches the size of solvated lithium ions, effectively preventing the infiltration of solvent molecules while facilitating the flow of lithium ions. This alteration by β-CD influences the formation of the SEI layer, leading to improved stability for Si nanoparticles. The implementation of the β-CD-based Si anode resulted in remarkable electrochemical performance with enhanced kinetics and stability. After 50 cycles at a current density of 500 mAhg^−1^, the anode demonstrated a high reversible capacity of 2562 mAhg^−1^, and even after 200 cycles at a current density of 1 A g^−1^, it retained a reversible capacity of 1944 mAhg^−1^. Moreover, the β-CD-based Si anode exhibited improved high-rate performance. This innovative strategy of utilizing β-CD as a lithium-ion diffusion channel offers a promising approach for designing high-capacity batteries with enhanced performance and stability for future generations of lithium-ion battery technology.

### 2.3. Cyclodextrin-Based Separators

**Figure 12:**
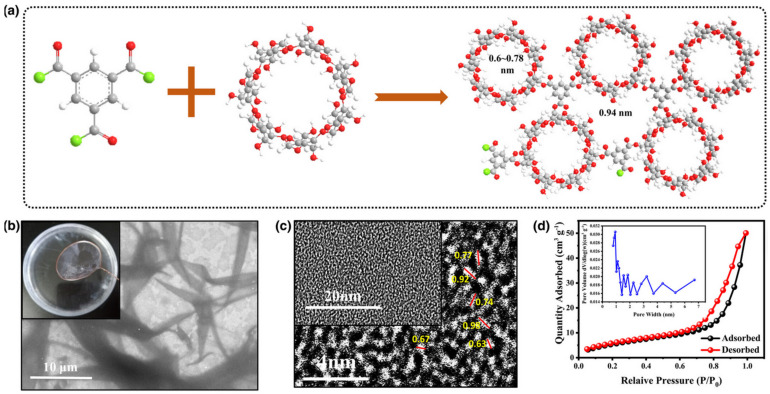
(**a**) Molecule structure model, (**b**) TEM and digital photo images, (**c**) HR-TEM image, and (**d**) N2 adsorption/desorption isotherm. Insert is pore-size distribution curve of β-CD-TMC molecular sieve film. Reproduced by permission [132].

Numerous efforts have been made to address the limitations of Li-S batteries [115,116]. For instance, one approach involves incorporating sulfur into highly conductive graphene and carbon nanotube frameworks to tackle its insulating nature [117,118]. The separator in the Li-S battery configuration plays a crucial role in key battery performances such as internal resistance, capacity, cycle property, and safety, as it prevents direct contact between the cathode and anode [119]. Presently, commercial polyolefin separators (polypropylene and polyethylene) are commonly used in Li-S batteries. However, these separators have drawbacks such as low porosity, poor electrolyte affinity, and inferior thermal stability, leading to issues like polysulfides’ “shuttle effect”. This effect results in the loss of the active sulfur substance, rapid capacity decay, and other problems [120,121]. To overcome these challenges, electrospun nanofiber membranes have proven to be more effective as separators for Li-S batteries due to their high porosity, large specific surface area, and chemical stability [121,122]. Moreover, the strong affinity between the electrospun nanofiber membrane and electrolyte significantly enhances lithium-ion migration [123,124]. In the past, electrospun cellulose-acetate membranes were used as separators in LIB because of their polar nature and strong affinity to the electrolyte [125]. Also, electrospun nanofiber membranes with oxygen-rich polar groups have been employed as separators in Li-S batteries to effectively inhibit the “shuttle effect” by forming a strong binding energy with polysulfides [126,127]. In addition to chemical adsorption, a physical barrier and electrostatic repulsion have also shown effectiveness in inhibiting the “shuttle effect” [128,129]. Due to the small size of polysulfides (1.2–1.7 nm), a microporous membrane is required to block polysulfide shuttling while enabling the free passage of lithium ions (0.152 nm) [130]. To address this challenge, Zhou et al. [131] synthesized metal-organic frameworks (MOFs) with a pore size of 0.9 nm as a molecular sieve to inhibit polysulfides. However, incorporating MOFs into separators increased the thickness and weight, leading to the reduced electrochemical performance of Li-S batteries. Furthermore, the accumulation and aggregation of MOFs could affect the blocking effect of polysulfides. Thus, a thin and lightweight molecular sieve membrane that effectively blocks polysulfides while facilitating the transfer of lithium ions is highly desirable for high-performance Li-S batteries. The small cavity diameter of β-CD (0.60–0.78 nm) is expected to inhibit polysulfide shuttling. Additionally, the numerous active hydroxyl functional groups on the outer surface of CD molecules provide many reaction sites for modifications and crosslinking, enabling the formation of a microporous membrane. Consequently, CD serves as an ideal building block to introduce an additional physical barrier onto electrospun nanofiber membranes, effectively preventing the “shuttle effect” in Li-S batteries. Shuanglin Wu et al reported a novel approach to address the “shuttle effect” in lithium-sulfur (Li-S) batteries (Figure 12) [132]. The method involves applying a thin molecular sieve film onto a recycled cellulose acetate (CA) membrane derived from cigarette filters. The film utilizes a truncated cone structure known as β-cyclodextrin (β-CD) to physically block and chemically trap polysulfides, while also significantly enhancing ion transport. Additionally, a layer of graphite carbon (C) is sputtered onto the β-CD-free side of the separator, facing the cathode, serving as an upper-current collector without adding much thickness. These modifications result in remarkable performance improvements, with an initial discharge capacity of 1378.24 mAh/g and excellent long-term cycling stability, retaining 863.78 mAh/g after 1000 cycles at 0.2 C. In comparison, the battery with the β-CD/CA/C separator outperforms the PP separator by more than three times after 500 cycles. Notably, the unique funnel-shaped channels of the β-CD induce a differential ionic fluid pressure on both sides of the separator, accelerating ion transport by up to 69%. Furthermore, the battery exhibits a 65.3% faster charging rate, reaching 9484 mA/g. This “funnel effect” of the separator offers a promising and efficient solution for rapid charging in Li-S batteries and other lithium secondary batteries.

## 3. Limitation

- **Scalability:** Although cyclodextrins have shown promising results at the laboratory level, it is still difficult to scale them up for mass battery production. Future studies should examine ways to guarantee the reliable and effective production of cyclodextrin-based products on a larger scale. To meet the requirements of high-volume battery production, this would entail creating scalable synthesis routes and optimizing manufacturing procedures.
- **Cost-effectiveness:** Another crucial factor to consider is the economic viability of cyclodextrin-based technologies. To compete with current battery materials, the price of cyclodextrins and the methods used in their synthesis should be reduced. To lower production costs and increase their commercial viability, future studies should investigate cost-effective manufacturing techniques, such as utilizing renewable feedstocks or enhancing the efficiency of cyclodextrin synthesis.
- **Recyclability:** Given the growing emphasis on sustainability and the principles of the circular economy, the ability to recycle battery parts is crucial. Materials based on cyclodextrin must be created with recycling in mind. Future studies should consider the recycling potential of cyclodextrin-based products and how they affect the entire battery-recycling process. Reducing waste and environmental impact would be helped by the development of cyclodextrin-based technologies that can be easily recycled or integrated into already-existing recycling processes.

## 4. Future Research Opportunities

To further enhance the performance of cyclodextrins in lithium batteries, future studies should focus on improving their structural and physical characteristics to increase capacity, enhance safety features, and improve cycling stability. Additionally, understanding how cyclodextrins interact with other battery components, such as electrodes and electrolytes, can contribute to overall battery performance. To assess the environmental advantages of cyclodextrin-based materials and identify areas for improvement, comprehensive life-cycle assessments (LCAs) are crucial. These assessments should quantify the environmental impact of manufacturing, using, and disposing of cyclodextrin-based batteries. These data will enable researchers to develop strategies for reducing the overall environmental impact and establish cyclodextrins as more environmentally friendly substitutes for traditional battery materials. Furthermore, further research should concentrate on optimizing the synthesis and functionalization of cyclodextrins, including sustainable and efficient production methods, exploring novel functionalization techniques for tailoring properties to specific battery applications, and examining potential synergistic interactions with other materials. Streamlining the manufacturing process of cyclodextrin-based battery systems can ultimately enhance their performance.

## 5. Conclusions

The host–guest chemistry of cyclodextrin (CD) has shown promise in enhancing lithium-ion battery (LIB) performance. Several issues with battery design and functionality have been solved by using CD-based materials. Li-based batteries perform better thanks to functional organic polymers developed using the CDs’ encapsulation properties. With their ideal size and ability to form hydrogen bonds, CD-based materials act as a lithium-ion diffusion channel and help to create the solid electrolyte interface (SEI) layer. CDs have also been used as solid electrolytes in solid-state batteries and as binders to ensure strong adhesion between electrode components. Electrolytes have been modified to enhance LIB performance using CD-based materials, silicon anode binders have been created, and separators have been developed. These applications of CD host–guest chemistry have the potential to advance battery technology and meet the increasing demand for effective energy-storage options.

## Figures and Tables

**Table 1:** Capacity, Capacity Retention, Inclusion Strength, and Cross-Linking Strength of a Composite Comprising α, β and γ CDp and Guest Molecules. Reproduced by permission [81].

Binder	Linker	ICE (%)	Capacity Retention	Inclusion	Cross-Linking
β-CD	-	78	69	no	no
**β-CD**	**6AD**	**84**	**90**	**Strong**	**Strong**
β-CD	1AD	83	23	Strong	no
α-CD	-	79	64	no	no
α-CD	6AD	84	19	no	no
α-CD	1AD	87	29	no	no
γ-CD	-	79	53	no	no
γ-CD	6AD	85	35	Weak	Weak
γ-CD	1AD	84	23	Weak	no

## Abbreviations

CD	Cyclodextrin
α-CD	Alpha Cyclodextrin
β-CD	Beta Cyclodextrin
γ-CD	Gama Cyclodextrin
C- β-CD	Carbonate beta cyclodextrin
LIB	Lithium Ion Battery
PVDF	Polyvinylidene fluoride
NMP	N-Methyl-1-2pyrrolidine
Li^+^	Lithium ions
Na^+^	Sodium ions
H^+^	Hydrogen ions
OH^-^	Hydroxide ions
e^−^	electron ions
FeF_2_	Iron(II) Fluoride
CoFe	A binary intermetallic compound made up of cobalt (Co) and iron (Fe)
LiCoO_2_	A layered oxide material made up of lithium (Li), cobalt (Co) and oxygen (O_2_)
Li_0.5_CoO_2_	A layered oxide material made up of lithium (Li), cobalt (Co) and oxygen (O_2_), 0.5″ in the formula refers to the stoichiometry of the compound, meaning there is half as much lithium as cobalt in the compound.
Li	Lithium
LiMn_2_O_4_ or (LMO)	A chemical compound made up of lithium (Li), manganese (Mn), and oxygen (O_4_). It is commonly referred to as Lithium Manganese Oxide (LMO)
Ti	Titanium
V	Vanadium
Cr	Chromium
Mn	Manganese
Fe	Iron
Co	Cobalt
Ni	Nickel
Cu	Copper
Mo	Molybdenum
W	Tungsten
Ru	Ruthenium
O	Oxygen
N	Nitrogen
F	Fluorine
S	Sulphur
Si	Silicon
Ge	Germanium
Sn	Tin
Al	Aluminium
Sb	Antimony
PAA	Polyacrylic acid
H_2_O_2_	Hydrogen peroxide
NH_4_VO_3_	ammonium vanadate salt
Li_2_CO_3_	Lithium Carbonate
V	Volt
Si β-CDp	Silicon anode using beta cyclodextrin polymer as binder
XRD	X-ray diffraction
TEM	Transmission electron microscope
SEM	Scanning electron microscope
CO_3_O_4_	Cobalt Oxide
mAh g ^−1^	milliampere-hours per gram
C	Capacity
GiTT	Galvanostatic intermittent titration technique
CV	Cyclic voltammetry
EIS	Electrochemical impedance spectroscopy
1D	one dimensional
°A	Angstrom
rGO	Reduced graphene oxide
CFP	Carbon Foam with Porous
C/S	Carbon and sulfur mixture
1D NN	one-dimension nanonets
1D-NP	one-dimension nanoparticle
TFSI	Bis (Trifluoromethylesulonyl)imide
SQM	Semi-empirical quantum mechanics
RMβCD	Randomly Methylated beta cyclodextrin
Li-S	lithium and sulfur
COSMO-RS:	Conductor like screening model for real solvent
1D	one dimensional
2D	Two dimensional
1H NMR	Proton nuclear magnetic resonance
DME	1,2-Dimethoxyethane
MeOH	Methanol
nm	Nanometer
Mβ-CD	Methyl-beta-cyclodextrin
PVP	Polyvinylpyrrolidone
ACS	Amylose corn starch
DFT	Density function theory
PEG	polyethylene glycol
PTMC	Trimethyl carbonate
CDTPEs	Cyclodextrin based triblock polymer electrolyte.
PEO	Poly(ethylene oxide)
SSE	Solid state electrolytes
ASSLB	All- Solid- Sate Lithium Batteries
LiTFSI	Lithium Trifluoromethanesulfonimide
CSE	Composite solid electrolyte
3D	Three dimensional
CMC	Carboxymethlcellulose
LZTO	Is a layer oxide material with chemical formula Li_2_ZnTi_3_O_8_
ECH	Epichlorohydrin
NS	nanosponge
Si-S	silicon and sulfur
CNS	Carbon nanosponges
